# YB1 associates with oncogenetic roles and poor prognosis in nasopharyngeal carcinoma

**DOI:** 10.1038/s41598-022-07636-z

**Published:** 2022-03-08

**Authors:** Yuting Zhan, Xianyong Chen, Hongmei Zheng, Jiadi Luo, Yang Yang, Yue Ning, Haihua Wang, Yuting Zhang, Ming Zhou, Weiyuan Wang, Songqing Fan

**Affiliations:** 1 Department of Pathology, the Second Xiangya Hospital, Central South University, Changsha, 410011 Hunan China; 2 Department of Pathology, Affiliated Hospital of Xiangnan University, Chenzhou City, 423000 Hunan China; 3 Cancer Research Institue Xiangya School of Medicine, Central South University, Changsha, 410078 Hunan China; 4 Department of Pathology, Xiangya Hospital of Central South University, Changsha, 410011 Hunan China

**Keywords:** Head and neck cancer, Oncogenes

## Abstract

Nasopharyngeal carcinoma (NPC) is the malignant tumor arising from the nasopharynx epithelium with ethnic and geographical distribution preference. Y-box binding protein-1 (YB1) is the highly expressed DNA/RNA-binding protein with cold shock domain, and enhanced YB1 expression was proved to be associated with many kinds of malignant tumors. There is no systematic study about the regulation of YB1 and cell proliferation, migration, invasion and stress granules (SGs) in NPC, and the relationship between YB1 expression and clinical characteristics and prognosis of NPC patients. We analyzed the mRNA expression of YBX1 in head and neck squamous carcinoma (HNSC) and NPC in databases, investigated the functions of YB1 in cell proliferation, migration and invasion and SGs formation of NPC cells, and detected expression of YB1 protein in a large scale of NPC samples and analyzed their association with clinicopathological features and prognostic significance of NPC patients. YBX1 mRNA was significantly high expression in HNSC and NPC by bioinformatic analysis, and higher expression of YBX1 mRNA indicated poorer prognosis of HNSC patients. Clinically, the expression of YB1 in NPC tissues was significantly higher than these in the control nasopharyngeal epithelial tissues. We further found that the expression of YB1 had an evidently positive relation with advanced clinical stages of patients with NPC. The overall survival rates (OS) were significantly lower for NPC patients with positive expression of YB1. Multivariate analysis confirmed that positive expression of YB1 was the independent poorer prognostic factor for patients with NPC. Moreover, compared with the immortalized nasopharyngeal epithelial cell line (NP69), the basal level of YB1 in NPC cell lines was significantly higher. Knocking down YB1 may inhibit Akt/mTOR pathway in NPC cells. Knocking down YB1 by small interfering RNAs can reduce the ability of proliferation, migration, invasion and SGs formation of NPC cells. The expression of YB1 in NPC cell lines or patients with NPC was significantly higher. The high expression of YB1 protein may act as one valuable independent biomarker to predict poor prognosis for patients with NPC. Knocking down YB1 may release the malignant phenotype of NPC cells.

## Introduction

Nasopharyngeal carcinoma (NPC) is the malignant tumor arising from the nasopharynx epithelium. Compared with other tumor types, NPC is confirmed with ethnic and geographical distribution preference, mainly in Southern China and Southeast Asian and North African countries^1^. Based on WHO criteria, NPC is classified into keratinizing squamous cell carcinoma and nonkeratinizing carcinoma, the latter of which is subdivided as differentiated and undifferentiated kinds. Almost all patients with non-keratinized nasopharyngeal carcinoma in South China are infected with Epstein-Barr virus^2^. It was estimated by Global Cancer Statistics 2018 that there would be about 129,079 and 72,987 new cases and cancer-related deaths of NPC, accounting for 0.7% and 0.8% of all new cancer patients and dead ones respectively^3^. Although early diagnosis and local control can be achieved, there are still some NPC individuals suffering from distant metastasis and poor prognosis.

Y-box binding protein-1 (YB1) is a member of the cold-shock domain protein superfamily, and it participates in many DNA/RNA‐dependent events such as DNA reparation, pre‐mRNA transcription and splicing, mRNA packaging and regulation of mRNA stability and translation^4^. High expression of YB1 has not only been found associated with a variety number of cancer such as laryngeal squamous cell carcinoma, non-small cell lung cancer (NSCLC), hepatocellular carcinoma, breast cancer, colorectal cancer^5–10^, also been reported to involve in the acquirement of chemoresistance of malignant tumors^11,12^. It was also reported that Ras GTPase-activating protein-binding protein 1 (G3BP1), one of the important components of stress granules (SGs), could be regulated by YB1 in human sarcomas, and YB1 could directly bind to and translationally activate the 5' untranslated region (UTR) of G3BP1 mRNAs, thereby controlling the formation of SGs^13^. The phosphorylation of eukaryotic translation initiation factor 2alpha (eIF2α) was considered as mechanisms that permit survival and recovery from stress, and was believed as the key process of SGs formation^14,15^. Our previous study has also verified that overexpression of G3BP1 associates with YB1 and p-AKT and predicts poor prognosis in NSCLC patients after surgical resection^9^.

Although many studies have been focused on roles of YB1 in the occurrence, development and treatment of malignant tumors, there is no research to investigate the relationship between the expression of YB1 and malignant phenotype as well as clinicopathological features for patients with NPC. In this study, we analyze the mRNA expression and potential clinical significance of YBX1 in patients with head and neck squamous carcinoma (HNSC) and NPC in TCGA database and GEO database respectively, also detected the expression of YB1 proteins in NPC tissues and control nasopharyngeal epithelial tissues by immunohistochemistry (IHC), seeking the relationship between expression of YB1 and clinicopathological features and their potential prognostic significance in NPC patients. We further investigate the roles of YB1 in cell proliferation, migration, invasion and SGs formation of NPC and its potential mechanism.

## Materials and methods

### Ethics statement

All samples were obtained with informed consent, and all protocols, specimen usage, and data retrieval were approved by the Ethics Review Committee of the Second Xiangya Hospital of Central South University (Scientific and Research Ethics Committee, No. Y202/2014). Written informed consent was obtained from all patients, and the written informed consent was obtained from the next of kin, caretakers, or guardians on the behalf of the minors/children participants involved in our study. All experiments were performed in accordance with relevant guidelines and regulations.

### Bioinformatics databases

ONCOMINE database: In our study, ONCOMINE database (www.oncomine.org) was used to analyze transcriptional expressions of YBX1 between various cancer tissues and their corresponding adjacent normal control samples. Difference of transcriptional expression was compared by students’t-test. Cut-off of *p* value and fold change were as follows: *p* value: 0.01, fold change: 1.5, gene rank: 10%, data type: mRNA.

UALCAN: UALCAN (http://ualcan.path.uab.edu) was used to analyze the mRNA expressions of YBX1 in HNSC and its corresponding adjacent normal control tissues; primary HNSC tissues and their association with clinicopathologic parameters was also analyzed by UALCAN. Difference of transcriptional expression was compared by students’ t test and *P* < 0.01 was believed as statically significant.

GEPIA: GEPIA (http://gepia.cancer-pku.cn/), a web server for cancer and normal gene expression profiling and interactive analyses, was used to analyze the mRNA expressions of YBX1 and prognosis of HNSC patients.

GEO database: Dataset GSE12452 was downloaded on the Gene Expression Omnibus (GEO, http://www.ncbi.nlm.nih.gov/geo/), which was used to analyze the mRNA expressions of YBX1 in NPC and its corresponding adjacent normal control tissues.

PathCards database: PathCards (https://pathcards.genecards.org/), an integrated database of human biological pathways and their annotations, was used to analyse YBX1 and its potentially involved pathways.

### Cell lines and cell culture

Cell lines used in this study were gifted by the Cancer Research Institute, Central South University. All cell lines were recently authenticated using short tandem repeat (STR) profiling by Microread Gene Technology (Beijing, China). The immortalized nasopharyngeal epithelial cell line (NP69) was cultured by defined keratinocyte-serum free medium (Gibco; Thermo Fisher Scientific, USA) at 37 °C and 5% CO_2_^16^. The human NPC cell lines (CNE2, HNE1, HNE2, 5-8F, 6-10B and HONE1) were cultured in DMEM medium (Biological Industries, Israel) supplemented with 10% fetal bovine serum (Biological Industries, Israel) at 37 °C and 5% CO_2_. These cell lines were immediately cultured and frozen down so that all cell lines could be restarted every 3–4 months from a frozen vial of the same batch of cells.

### Small interfering RNAs and transfection

The small interfering RNAs (siRNAs) and the control siRNA (siNC) were purchased by RiboBio Technology, Guangzhou, China. The following siRNAs were generated: siYB1-1: GACGGCAATGAAGAAGATAA; siYB1-2: GTTCAATGTAAGGAACGGAT; and siYB1-3: GGTTCCCACCTTACTACAT. The siNC was secretive and not public by company. Transfection was performed with 70 nM siRNAs per sample using polyplus jetPRIME transfection reagent (Polyplus, France).

### CCK8 assay and colony formation

Cell survival rates were estimated by the CCK-8 assay (Bimike, USA). After 24 h of transfection, 10^3^ cells were counted and seeded in 96-well plates with 100 μl medium each well. Each well was incubated with 10 μl CCK-8 solutions for 2 h away from light before measuring the absorbance at 450 nm by Multilabel Plate Reader (SpectraMax iD3, Molecular Devices). For colony formation assay, 2*10^3^ 5-8F cells and 3*10^3^ HNE2 cells were counted and seeded in 6-well plates after 24 h of transfection respectively, cells were fixed and stained with 0.1% crystal violet staining solution to visualize colonies after about 14 days. Experiments were performed in triplicate.

### Wound healing assay

When the transfected cells seeded in 6-well plate reached a confluent state, scratches were made using a sterile 10 μl pipette tip. The cells were then incubated with FBS-free culture medium. Images of the scratches were captured at 0, 24 and 48 h with Olympus CKX53 inverted microscope at 100 × magnification. The width of the scratch was analyzed using the Image Pro Plus6.0.

### Matrigel invasion assay

Transwell migration assay was performed using transwell inserts (3422, Costar, USA) with a filter of 8 μm pore. 7*10^4^ transfected cells were counted and seeded in the upper chamber of the insert (coated with 30ul matrigel 3 h prior to seeding cells), while the complete medium with 20% serum was added to the lower chamber. After 48 h incubation, the cells were fixed with formalin and stained with 0.1% crystal violet staining solution. Cells on the top surface of the membrane were wiped off, and cells on the lower surface were examined with Olympus CKX53 inverted microscope at 100 × magnification. The number of migrated cells was used as a measure of migration capacity.

### Induction of stress granules and immunofluorescence staining

Cells (1 × 10^4^) were planted on specialized 96-well plates for 24 h, and arsenic trioxide (ATO) was added to a final concentration of 500 μM for 1 h so as to induce SGs. Fixed with 4% paraformaldehyde for 60 min and blocked by normal goat serum for 30 min at room temperature. The wells of palate were incubated with mouse monoclonal antibodies against G3BP1 (Ras GTPase-activating protein-binding protein 1, Catalog: sc-365338, Santa Cruz Biotechnology) and rabbit monoclonal antibodies against YB1 (Catalog: #4202, Cell Signaling Technology) overnight, then stained with HRP goat anti-mouse IgG-R (Dylight 488, Goat Anti-Rabbit IgG, Catalog: #A23220, Abbkine) or HRP goat anti-rabbit IgG-R (Dylight 594, Goat Anti-Mouse IgG, Catalog: #A23410, Abbkine). Images were obtained with High content cell imaging analysis system.

### Patient cohorts

In this study, 410 cases of paraffin-embedded NPC including 302 males and 108 females were selected randomly from the files of the Department of Pathology, the Second Xiangya Hospital of Central South University (Changsha, China). All patients with NPC had been followed, some for as long as 10 years, during the period from January 2000 to December 2009. All specimens had been confirmed by pathological diagnosis according to the World Health Organization histological classification of NPC. The staging classification of the current analysis was carried out based on the criteria of the 8th edition of the AJCC/UICC TNM staging system of NPC. None of patients had previously been treated with radiotherapy or chemotherapy at the time of original biopsy. We also collected 53 cases of control nasopharyngeal epithelial tissues. Complete clinical records and follow-up data were available for all patients. Detailed cohort description was showed in Table 1.

**Table 1:** Clinicopathological features of patients with NPC and non-cancerous nasopharyngeal epithelial tissues.

Patients features	No. of patients (%)
**NPC patients**	
**Gender**	
Male	302 (73.7)
Female	108 (26.3)
**Age**	
< 40	86 (21.0)
≥ 40	324 (79.0)
**Clinical stages**	
I	4 (1.0)
II	113 (27.6)
III	166 (40.5)
IV	127 (31.0)
**LN status**	
No LNM	107 (26.1)
LNM(N1/N2/N3)	303 (73.9)
**Histological type**	
UDNPC	384 (93.7)
DNPC	26 (6.3)
**Survival status**	
Alive	321 (78.3)
Dead	89 (21.7)
**Non-cancerous nasopharyngeal epithelial tissues**	
**Gender**	
Male	33 (62.3)
Female	20 (37.7)
**Age**	
< 40	11 (20.7)
≥ 40	42 (79.2)

*DNPC* differentiated non-keratinized nasopharyngeal carcinoma, *UDNPC* undifferentiated non-keratinized nasopharyngeal carcinoma, *LN* lymph node, *LNM* lymph node metastasis.

### Immunohistochemical staining and scoring

The IHC staining for YB1 proteins was carried out by HRP-Polymer anti-Mouse IHC Kit. The staining conditions for each antibody were followed and adjusted according to our laboratory experience^9,17,18^. 1:200 dilution of primary antibody to YB1 (Monoclonal Rabbit antibody, Catalog: #4202, Cell Signaling Technology) was applied in this study. Positive control slides were included in every experiment in addition to the internal positive controls. The specificity of the antibody was confirmed with matched IgG isotype antibody as a negative control.

YZ and HZ made a score for each slide independently, both of whom were blinded to the clinicopathological data at 200 × magnification light microscopy. Evaluation was based on the intensity and extent of staining, and a semi-quantitative evaluation for YB1 was performed using the method described as follows: Staining intensity for each antibody was scored as 0 (negative), 1 (weak), 2 (moderate), and 3 (strong). Staining extent was scored as 0 (no staining), 1 (1–25%), 2 (26–50%), 3 (51–75%), and 4 (76–100%), which depended on the percentage of stained cells. The staining intensity and extent were both based on tumor cells rather than lymphocytes. YB1 expression was calculated by multiplying the intensity score by tumor staining extent (0, 1, 2, 3, 4, 6, 8, 9 and 12). Referred to published literatures and combined with our practical situation, we made optimal cut-off levels as follows^19^. As for YB1, a staining index score of ≤ 2 was used to define tumors with negative expression while ≥ 3 indicated positive expression. Agreement between the two evaluators was 95%, and all scoring discrepancies were resolved through discussion between the two evaluators.

### Quantitative RT-PCR (qRT-PCR) analysis

Total RNA was extracted using TRIzol reagent (Accurate Biotechnology, China) and 1 µg of total RNA was used for synthesis of first-strand cDNA with a reverse transcriptase kit (Accurate Biotechnology, China). The mRNA level was measured by qPCR with Kit AG11701 (Accurate Biotechnology, China) on a Real-time fluorescence quantitative PCR system (Bio-Rad, USA). The primers used in the study were as follows: YBX1 Forward: AAGGAGAAAAGGGTGCGGAG; YBX1 Reverse: CCTACGACGTGGATAGCGTC; GAPDH Forward: CGAGATCCCTCCAAAATCAA; GAPDH Reverse: TTCACACCCATGACGAACAT. GAPDH was used as control. Relative expression was determined with GAPDH control through the 2^−∆∆Ct^ method.

### Western blotting

Protein lysates preparation and western blot analysis were performed as previously described^20^. Specifically, the primary antibodies were applied as follows: rabbit monoclonal antibodies against YB1 (Monoclonal Rabbit antibody, Catalog: CY5462, Abways) for 1:2000. Rabbit polyclonal antibodies against E-cadherin (Polyclonal Rabbit antibody, Catalog: 20,874–1-AP, Proteintech) for 1:4000. Mouse monoclonal antibodies against N-cadherin (Monoclonal mouse antibody, Catalog: 66,219–1-Ig, Proteintech) for 1:1000. Mouse monoclonal antibodies against Vimentin (Monoclonal mouse antibody, Catalog: BF8006, Affinity) for 1:1000. Rabbit monoclonal antibodies against phospho-eIF2α (Ser51) (Monoclonal Rabbit antibody, Catalog: #3398, Cell Signaling Technology) for 1:1000. Rabbit monoclonal antibodies against eIF2α (Monoclonal Rabbit antibody, Catalog: #5324, Cell Signaling Technology) for 1:2000. Rabbit monoclonal antibodies against phospho-mTOR (Ser2448) (Monoclonal Rabbit antibody, Catalog: #2976, Cell Signaling Technology) for 1:1000. Mouse monoclonal antibodies against mTOR (Monoclonal mouse antibody, Catalog:66888-1-Ig, Proteintech) for 1:1000. Rabbit monoclonal antibodies to Akt1 (phospho S473) (Monoclonal Rabbit antibody, Catalog: ab81283, Abcam) for 1:3000. Rabbit polyclonal antibodies against Akt1 (Polyclonal Rabbit antibody, Catalog: #9272, Cell Signaling Technology). Rabbit monoclonal antibodies against phospho-S6 (Ser240/244) (Monoclonal Rabbit antibody, Catalog: #5364, Cell Signaling Technology) for 1:2000. Mouse monoclonal antibodies against S6 (Monoclonal mouse antibody, Catalog: 66886-1-Ig, Proteintech). Mouse monoclonal antibodies against GAPDH (Monoclonal mouse antibody, Catalog: 60004-1-Ig, Proteintech) for 1:50000.

### Statistical methods

Statistical analyses were performed by log-rank test, Chi-square test, multivariate Cox regression analysis, and student’s t-test as appropriate using SPSS for Windows (18.0; SPSS, Inc.) and GraphPad Prism (Prism 5.0; GraphPad Software Inc.) packages. A *P* value < 0.05 was considered significant. Error bars indicate the standard deviation in all the Figures. *P < 0.05, **P < 0.01, ***P < 0.001 by two-tailed t-test.

## Results

### Significant higher expression of YB1 in patients with head and neck cancer or NPC by bioinformatic analysis

**Figure 1:**
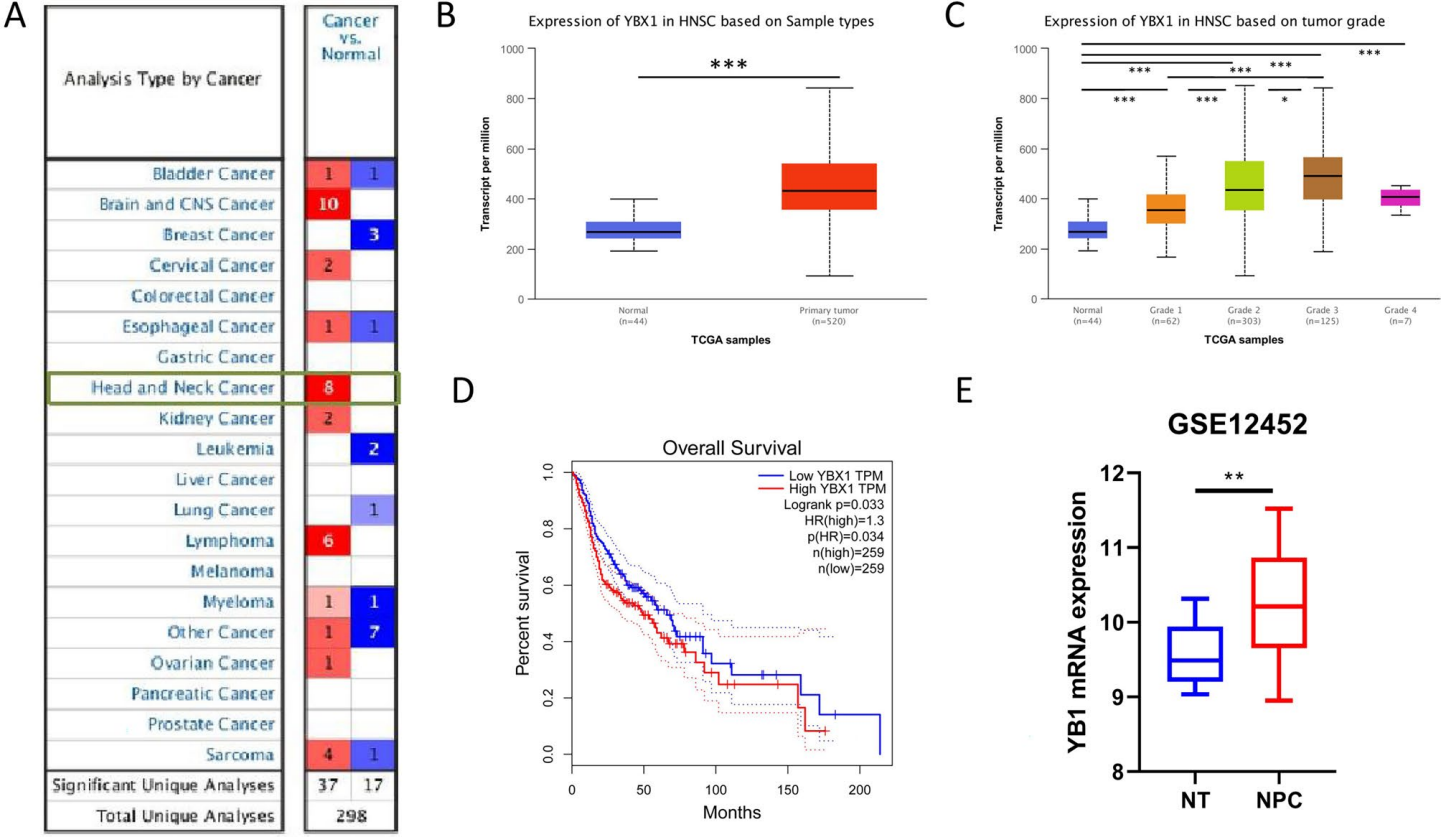
Significant higher expression of YBX1 mRNA in patients with head and neck cancer (HNSC) and nasopharyngeal carcinoma (NPC) by bioinformatic analysis. (**A**) Transcriptional expression of YBX1 in 20 types of cancer (ONCOMINE database); (**B**) mRNA expression of YBX1 in HNSC tissues and corresponding normal tissues (UALCAN database); (**C**) Relationship between mRNA expression of YBX1 and tumor grade of HNSC patients (UALCAN database); (**D**) Prognostic value of mRNA expression of YBX1 in patients with HNSC (GEPIA database); (**E**) Transcriptional expression of YBX1 in NPC tissues and corresponding normal tissues (GEO database, GSE 12,452).

NPC is one of the HNSC with ethnic and geographical distribution preference; therefore, we searched the differential expression of YBX1 mRNA in 20 kinds of malignant tumors including HNSC by ONCOMINE database. As shown in Fig. 1A, the majority indicated the higher expression of YBX1 mRNA in tumors (red boxes) than the controls (blue boxes) including HNSC. The specific parameters of differential expression of YBX1 in HNSC of ONCOMINE database were shown in supplementary Table 1. In UALCAN database, mRNA expression of YBX1 also was also found to be up-regulated in HNSC tissues compared to the control ones (Fig. 1B). Further analysis showed that mRNA expression of YBX1 associated with tumor grade by UALCAN, and mRNA expression of YBX1 seemed to be higher with the increase of tumor grade (Fig. 1C). Due to the small sample size of grade 4 (only 7 samples), the highest mRNA expression of YBX1 was in grade 3. We also evaluated the influence of YBX1 on clinical prognosis using Gene Expression Profiling Interactive Analysis (GEPIA). In Fig. 1D, higher expressions of YBX1 were found to associate with poorer OS in HNSC patients than lower ones. The specific parameter is HR = 1.3 and p = 0.033. Since NPC is one of type of HNSC, we downloaded dataset GSE12452 on GEO database and found that the expression of YBX1 mRNA was higher in NPC than the corresponding normal ones (Fig. 1E).

### Expression of YB1 correlated with clinicopathological features and predicted overall survival of patients with NPC

**Figure 2:**
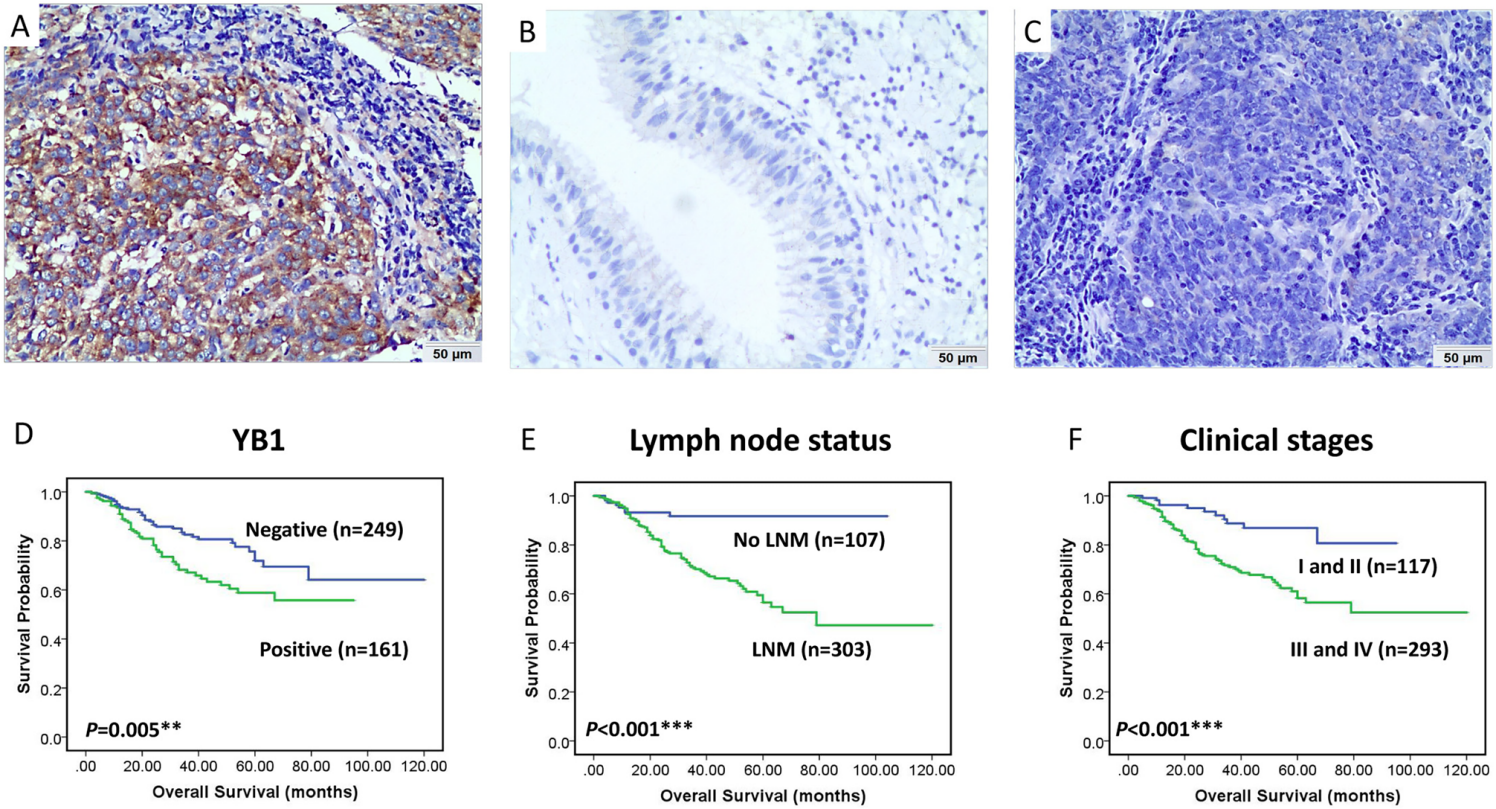
Expression of YB1 predicted overall survival of patients with NPC. (**A**) Strong positive expression of YB1 protein was presented in the NPC; (**B**) weak positive expression of YB1 was indicated in the columnar epithelial cells of non-cancerous nasopharyngeal tissue; (**C**) the matched IgG isotype antibody as a negative control showed no positive staining of YB1 in the NPC; (**D**) The overall survival rates were significantly lower for NPC patients with positive expression of YB1 (*P* = 0.005); (**E**) the overall survival rates were significantly lower for NPC patients with lymph node metastasis (*P* < 0.001); (**F**) The overall survival rates were significantly lower for NPC patients with advanced stages (stage III and IV) than early stages (stage I and II) (*P* < 0.001).

We detected the expression and cellular location of YB1 in NPC and the non-cancerous nasopharyngeal epithelia tissues by IHC. The results showed that the membrane and cytoplasma of cancer cells was diffusely stained for YB1, whereas nuclear staining was rarely identified. Strong positive expression of YB1 protein was presented in the NPC (Fig. 2A) and weak positive expression of YB1 was indicated in the columnar epithelial cells of non-cancerous nasopharyngeal tissue (Fig. 2B). The matched IgG isotype antibody as a negative control showed no positive staining of YB1 in the NPC (Fig. 2C). Among NPC and the non-cancerous nasopharyngeal epithelia tissues included in this study, 39.3% (161/410) and 1.9% (1/53) demonstrated positive expression of YB1 in NPC and non-cancerous nasopharyngeal epithelia tissues respectively, and there was a significant difference between NPC and the non-cancerous nasopharyngeal epithelia tissues in the expression of YB1 (*P* < 0.001).

Furthermore, we investigated the associations between expression of YB1 protein and clinicopathological features of NPC including gender, age, clinical stages, lymph node status and histological type in univariate Chi Square Test. It can be seen from the data in Table 2 that the expression of YB1 had an evidently positive relation with advanced clinical stages of patients with NPC (*P* = 0.014), but there was no relation could be seen in terms of gender, age, lymph node status and histological type (all *P* > 0.05).

**Table 2:** Analysis of association between expression of YB1 protein and clinicopathological features of NPC.

Clinicopathological features	YB1		
	P (%)	N (%)	*P value*
**Gender**			0.434
Male (n = 302)	122 (29.8)	180 (43.9)	
Female (n = 108)	39 (9.5)	69 (16.8)	
**Age**			0.760
< 40 (n = 86)	35 (8.5)	51 (12.4)	
≥ 40 (n = 324)	126 (30.7)	198 (48.4)	
**Clinical stages**			*0.014
I and II (n = 117)	35 (8.5)	82 (20.0)	
III and IV (n = 293)	126 (30.7)	167 (40.8)	
**LN status**			0.355
No LNM (n = 107)	38 (9.3)	69 (16.8)	
LNM (n = 303)	123 (30)	180 (43.9)	
**Histological type**			0.931
UDNPC (n = 384)	151 (36.8)	233 (56.8)	
DNPC (n = 26)	10 (2.4)	16 (4.0)	

*DNPC* differentiated non-keratinized nasopharyngeal carcinoma, *UDNPC* undifferentiated non-keratinized nasopharyngeal carcinoma, *LN* lymph node, *LNM* lymph node metastasis, *P* positive, *N* negative.

*χ^2^, *P* < 0.05 (2-tailed).

To further examine the impact of YB1 expression on the overall survival rates (OS) of NPC patients, we employed the Kaplan–Meier analysis to plot the survival curve, and statistical significance was assessed using the log-rank test. Univariate survival analysis (log-rank test) showed that the OS rates was significantly lower for NPC patients with positive expression of YB1 (*P* = 0.005, Fig. 2D). We also analyzed the survival rates of patients with NPC according to the conventional prognostic parameters including lymph node status and clinical stages by univariate survival analysis. Compared with patients without lymph node metastasis, lower OS rates could be seen for those with lymph node metastasis in NPC patients (*P* < 0.001, Fig. 2E). Besides, it was showed that the patients with advanced-stages (clinical stage III and IV) had lower OS rates than those with early-stages (clinical stage I and II) (*P* < 0.001, Fig. 2F ).

Multivariate Cox proportional hazard regression analysis was also carried out to further investigate whether the positive expression of YB1 protein was the independent prognostic factor for NPC patients (Table 3). In multivariate analysis of the features of patients with NPC, which included gender, age, histological type, LNM status, clinical stages, YB1 expression, positive expression of YB1 was identified as independent poorer prognostic factors for patients with NPC (*P* = 0.021), as well as lymph node metastasis, advanced clinical stages and gender (*P* = 0.002, *P* = 0.022 and *P* = 0.026 respectively). There were no prognostic effects detected in NPC patients including age and histological type (both *P* > 0.05).

**Table 3:** Summary of multivariate of Cox proportional regression for overall survival in 410 cases of NPC.

Parameter	SE	Wald	Sig	Exp (B)	95.0% CI for Exp (B)	
					Lower	Upper
Gender	0.272	4.987	0.026*	0.545	0.320	0.928
Age	0.261	0.183	0.669	0.894	0.536	1.491
Histological type	0.425	0.109	0.742	0.869	0.378	1.999
LNM status	0.389	9.261	0.002**	0.306	0.143	0.656
Clinical stages	0.340	5.283	0.022*	0.457	0.235	0.891
YB1	0.215	5.292	0.021*	0.610	0.401	0.929

Multivariate analysis of Cox regression, *P < 0.05; ** *P* < 0.01.

CI, confidence interval; Exp (β), odds ratio; LNM, lymph node metastasis.

### Knock down of YB1 inhibit Akt/mTOR signaling pathway

**Figure 3:**
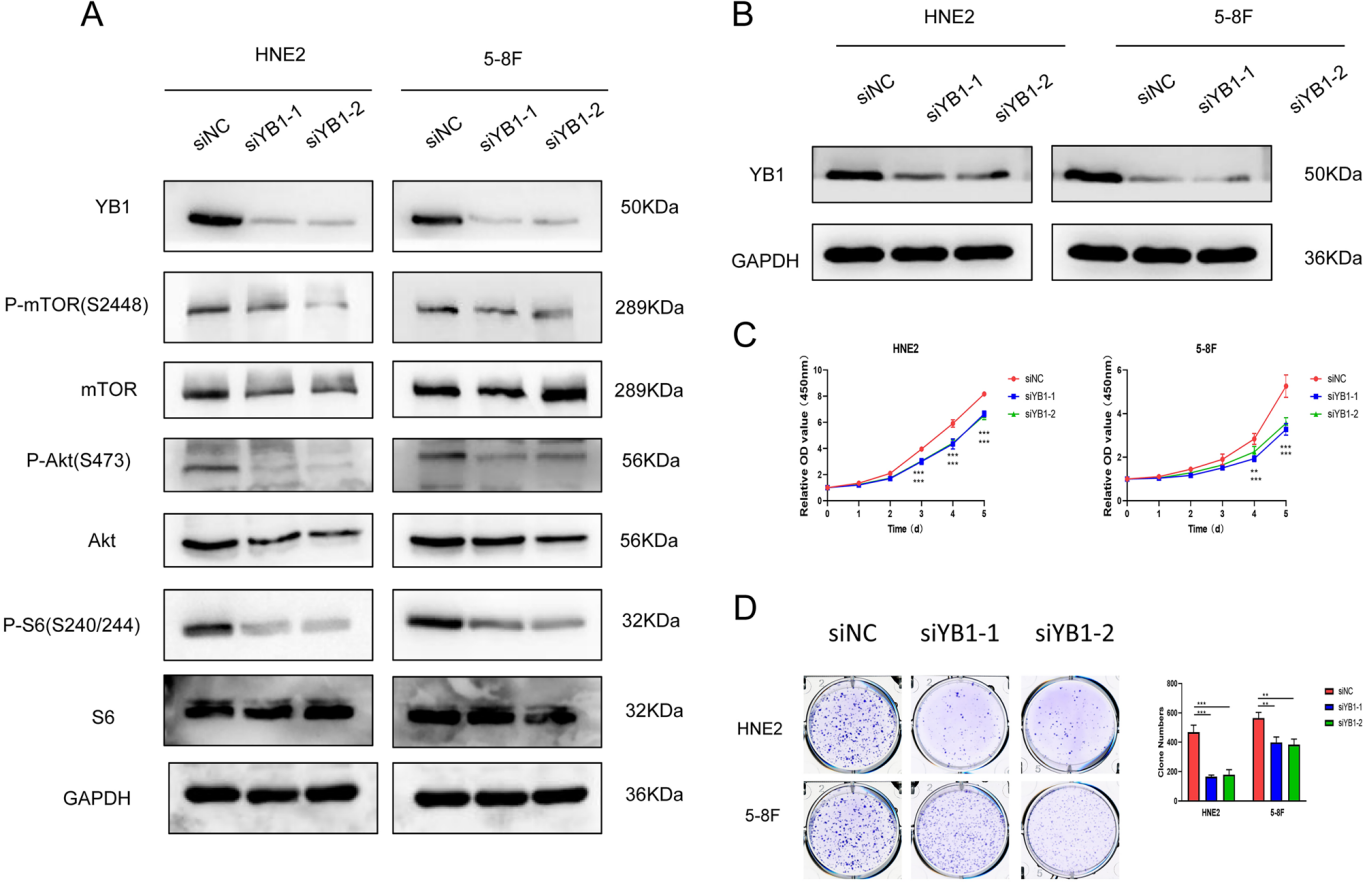
Knock down of YB1 inhibited Akt/mTOR signaling pathway and reduced NPC cell growth in vitro. (**A**) Knockdown of YB1 significantly inhibited AKT/mTOR signaling pathway. (**B**) The efficacy of knocking-down YB1 in HNE2 and 5-8F cell. (**C**) Knockdown of YB1 significantly inhibited the cell growth of HNE2 and 5-8F by CCK8 assay. (**D**) Knockdown of YB1 significantly inhibited the cell growth of HNE2 and 5-8F colony formation assay.

Pathcards (https://pathcards.genecards.org/), an integrated database of human biological pathways and their annotations, was used to analyse YBX1 and its potentially involved pathways (supplementary Fig. 2C). It was supposed that YBX1 was involved in Akt/mTOR pathway, and our experiment further verified it. Knocking down YB1 may reduce phosphorylation of Akt1, mTOR and S6 in specific site in HNE2 and 5-8F cells (Fig. 3A).

### Knock down of YB1 inhibited NPC cell growth, migration and invasion in vitro

The basal expression of YBX1 mRNA of all NPC cell lines (CNE2, HNE1, HNE2, 5-8F, 6-10B and HONE1) available was higher than that in immortalized nasopharyngeal epithelial cell line (NP69). We chose HNE2 and 5-8F with the moderate expression for the following study (supplementary Fig. 1A). To explore the influence of YB1 expression on tumor cell growth, three siRNAs were designed and verified, and siYB1-1 and siYB1-2 were used for the following experiments (supplementary Fig. 1B and C). Knockdown of YB1 significantly inhibited the cell growth of HNE2 and 5-8F by CCK8 assay (Fig. 3B, C), and the colony formation assay showed the same results (Fig. 3D).

**Figure 4:**
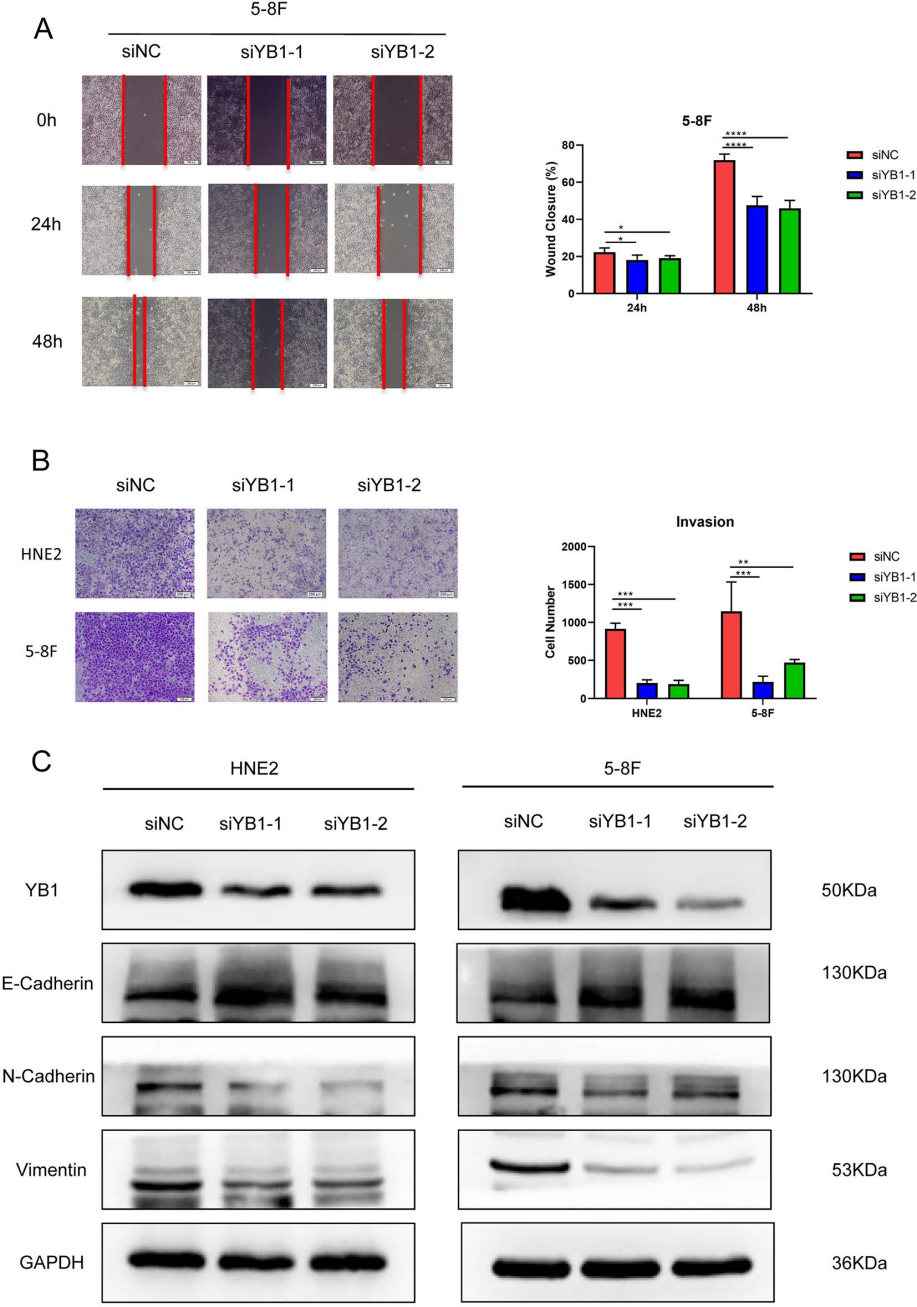
Knock down of YB1 inhibited NPC cell migration and invasion in vitro. (**A**) 5-8F cells with YB1 knock-down showed significantly lower migration rates in wound healing assay; (**B**) HNE2 and 5-8F cells with YB1 knock-down showed significantly lower invasion rates in matrigel invasion assay when compared with control cells. (**C**) Knocking down YB1 upregulated expression of E-Cadherin and downregulated expression of N-Cadherin and Vimentin.

Besides, cell with YB1 knock-down showed significantly lower migration rates in wound healing assay (Fig. 4A and supplementary Fig. 2A) as well as lower invasion rates in matrigel invasion assay when compared with control cells (Fig. 4B). Epithelial-interstitial transition (EMT) markers were also detected by WB, indicating that knocking down YB1 up-regulated expression of E-Cadherin and down-regulated expression of N-Cadherin and Vimentin (Fig. 4C).

### YB-1 recruitment to stress granules

**Figure 5:**
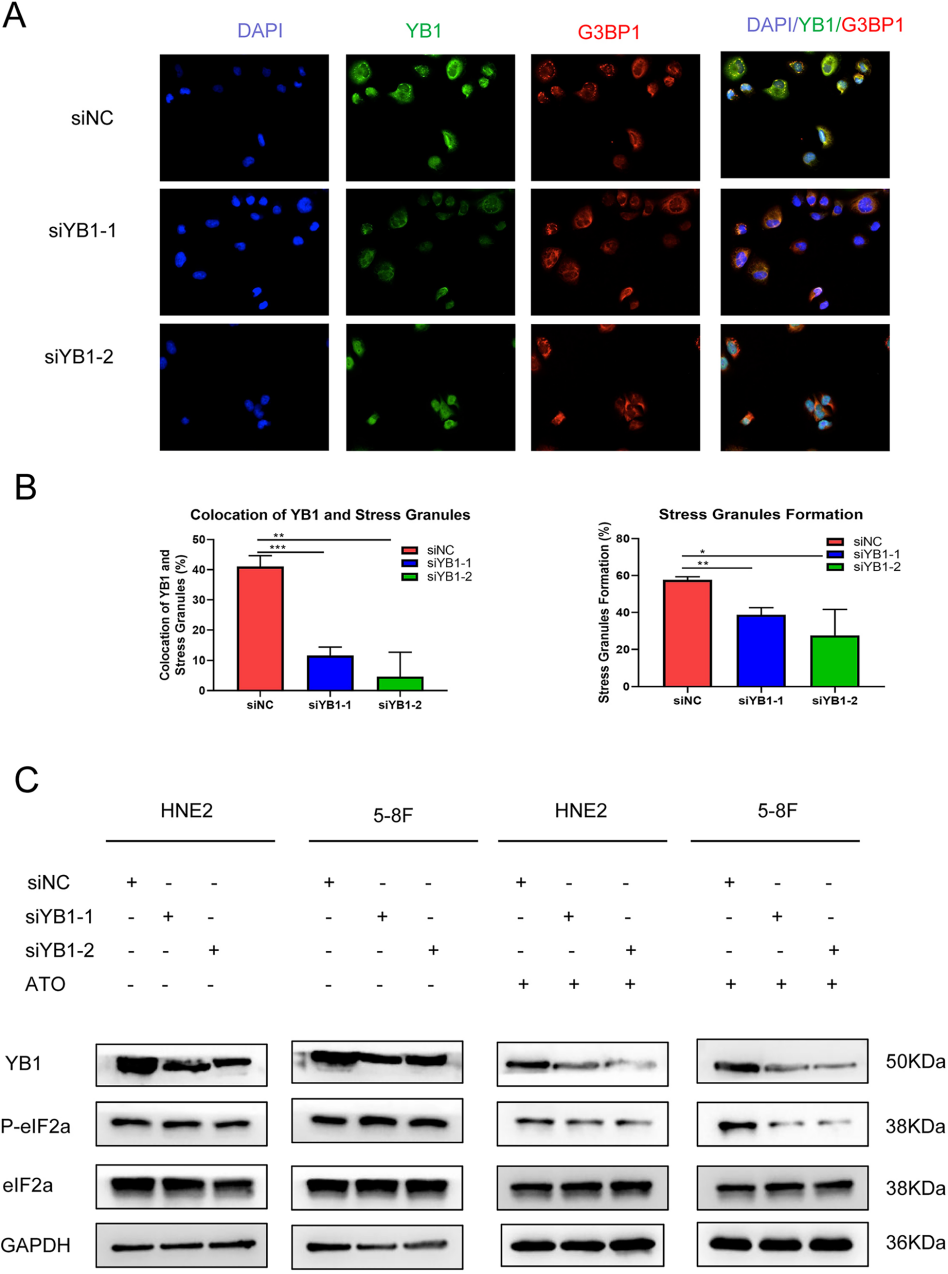
YB-1 recruitment to stress granules. (**A**, **B**) The detailed colocalization of YB1 protein and G3BP1 protein (G3BP1 was the component of SGs and was always regarded as the marker for SGs formation) in the presence of arsenic trioxide (ATO) stimulus with or without knocking down YB1. (**C**) Knocking down YB1 downregulated expression of phospho-eIF2α in the presence of ATO stimulus.

YB1, a kind of RNA-binding protein, was reported to colocalize to SGs, and somewhile to regulate the components of SGs such as G3BP1 in mesenchymal tissue-derived tumors such as Ewing sarcoma and osteosarcoma^13,21^. In this study, we detected the colocalization of YB1 protein and G3BP1 protein (the component of SGs and was always regarded as the marker for SGs formation) in the presence of ATO stimulus (Fig. 5A). Interestingly, the reduction of SGs formation (the granules of G3BP1 in cytoplasma) could be observed when knocking down YB1 protein with specific siRNAs (Fig. 5B and supplementary Fig. 2B). Since the reduction of phospho-eIF2α was regarded as typical mechanism for SGs formation, we detected phospho-eIF2α and eIF2α in the presence or without presence of ATO stimulus. Knocking down YB1 could significantly reduce phospho-eIF2α expression after ATO stimulus, however, this phenomenon didn’t exist without ATO stimulus (Fig. 5C).

## Discussion

YB1 is the highly expressed RNA-binding protein with cold shock domain. YB1 was found to associate with about 20 percent of cellular mRNAs and might be targets of YB1-mediated regulation at the level of translation or stability^22^. Almost every normal tissue was detected to express YB1 protein, and enhanced YB1 expression was proved to associate with occurrence, development and prognosis of malignant tumors, as well as response to chemotherapy. It was reported that higher expression of YB1 protein correlated with advanced T stage, poor differentiation and cervical metastasis in laryngeal squamous cell carcinoma patients, and YB1 could promote proliferation, invasiveness and migration of Hep-2 cells in vitro^5^. In the research of lung adenocarcinoma, YB1 is positively associated with TNM stages and cancer differentiation and patients with high expression of YB1 had poorer survival outcomes, and deeper study revealed that metastasis associated in colon cancer-1 (MACC1) was the target of YB1^6^. Another study about lung adenocarcinoma showed the similar results, and it revealed in vitro study that YB1 expression was closely related to cell cycle progression, cell proliferation apoptosis via the CDC25a pathway^23^. Besides, it was verified that YB1 could promote hepatic cells proliferation, migration, and drug-resistance, which was supposed to be involved in Wnt/β-catenin signaling pathway^7^. Moreover, YB1 was found to be overexpressed in pancreatic cancer patients, and high YB1 expression level was correlated with perineural invasion^24^. Apart mentioned above, YB1 expression level was still confirmed to relate to occurrence, development and prognosis of breast cancer, colon cancer, renal cell carcinoma and cervical cancer^8,25–28^, as well as drug resistance to chemotherapy^11,29–31^. More and more evidence indicated that nuclear localization and/or overexpression of YB1 predicted severe malignant phenotypes and poor outcomes for patients^32,33^.

In fact, the subcellular location of YB1 was closely related to its phosphorylation at Ser102 and intracellular RNA distribution^34^. The nuclear translocation of YB1 was believed to associate with stemness of cancer cells and conferred drug resistance, and inhibiting this process could reduce the P-gp expression and reverse the chemoresistance of resistant cancer cells^30,35^. Apart its nuclear translocation, the overexpression of YB1 in cytoplasm also played oncogenic roles. One of the important functions was the regulation of SGs formation. It was reported that YB1 could directly bind to tiRNAs via its cold shock domain, which was required for packaging of tiRNA-repressed mRNAs into SGs^36^. Besides, Somasekharan SP *et.al* demonstrated that YB1 could directly bind to and translationally activate the 5' untranslated region of G3BP1 mRNAs, and thereby controlled the availability of the G3BP1 SG nucleator for SG assembly^13^. As DNA/RNA binding protein, YB1 was proved to influence EGFR expression apart from its classic roles in cell proliferation, migration and apoptosis^37^. The crosstalk between cytoplasmic YB1 and PI3K/Akt/mTOR signaling pathway was also believed to play oncogenic roles or contributed to chemoresistance^9,10,38^. In fact, PI3K/Akt/mTOR signaling pathway was regarded as a potential therapeutic target in head and neck cancer including NPC^39–41^, and activated PI3K/Akt/mTOR signaling might induce proliferation, migration and invasion in NPC^42,43^. Moreover, it was speculated that PI3K signaling could control stress granule assembly in a hierarchical manner, while rapamycin complex 1 (mTORC1) was found initially inactivated due to the formation of SGs. Various evidence hinted the relationship among YB1 expression, Akt/mTOR signaling activation and malignant phenotypes of cancer^44,45^.

In our study, we found that YB1 was mainly located in the membrane and cytoplasm of NPC cells, and knocking down YB1 expression could reduce the ability of cell proliferation, migration and invasion. Besides, it was observed that YB1 can colocalized into SGs when confronted with ATO stimulus, which might involve in phosphorylation of eIF2α, although repeated experiments showed that YB1 couldn’t regulate G3BP1 expression in NPC cells. We preliminarily established the relationship between YB1 and Akt/mTOR signaling, and knock down of YB1 might reduce Akt/mTOR pathway, which perhaps explain the phenotypes of proliferation, migration, invasion and SGs formation. Moreover, we showed that YB1 was highly expressed in NPC tissues compared with non-cancerous nasopharyngeal epithelia tissues, and it had an evidently positive relation with advanced clinical stages of patients with NPC. Kaplan–Meier analysis and log-rank test showed that the OS rate was significantly lower for NPC patients with positive expression of YB1.

In summary, we analyzed the mRNA expression of YBX1 in HNSC and NPC in databases, verified the functions of YB1 in NPC cells, explored its potential involved pathway, and detected expression of YB1 protein in a large scale of NPC samples. In the future, it is worthy to seek for the deeper mechanism for YB1 to regulate SGs formation in NPC cells and avoid adverse conditions; besides, it is important to explore whether YB1 would translocate into nucleus under certain situation. In the meantime, it was also potential for the combination between YB1 expression or inhibition and hot topics of tumor therapy, such as anti-EGFR, anti-PD-1 or anti-PDL-1.

## Supplementary Information

Supplementary Information.

Supplementary Figure 1.

Supplementary Figure 2.

Supplementary Table 1.
