# Supplementary material for: YB1 associates with oncogenetic roles and poor prognosis in nasopharyngeal carcinoma
Source: Sci Rep. 2022 Mar 8;12:3699. doi: 10.1038/s41598-022-07636-z (PMC8904596; doi:10.1038/s41598-022-07636-z)
Supplement: Supplementary file 1 — Supplementary Information. [file 41598_2022_7636_MOESM1_ESM.docx]

**Supplementary Figure 1:** (A) Compared with the immortalized nasopharyngeal epithelial cell line (NP69), the basal level of YB1 in NPC cell lines (CNE2, HNE1, HNE2, 5-8F, 6-10B and HONE1) was significantly higher; (B and C) three siRNAs were designed and verified in HNE2 (B) and 5-8F (C), and siYB1-1 and siYB1-2 were used for the formal experiments.

**Supplementary Figure 2:** (A) HNE2 cells with YB1 knock-down showed significantly lower migration rates in wound healing assay; (B) The global colocalization of YB1 protein and G3BP1 protein in the presence of [arsenic](javascript:;) [trioxide](javascript:;) (ATO) stimulus with or without knocking down YB1; (C) YB1 and its involved pathways.
