# Supplementary figures and images for: YB1 associates with oncogenetic roles and poor prognosis in nasopharyngeal carcinoma
Source: Sci Rep. 2022 Mar 8;12:3699. doi: 10.1038/s41598-022-07636-z (PMC8904596; doi:10.1038/s41598-022-07636-z)

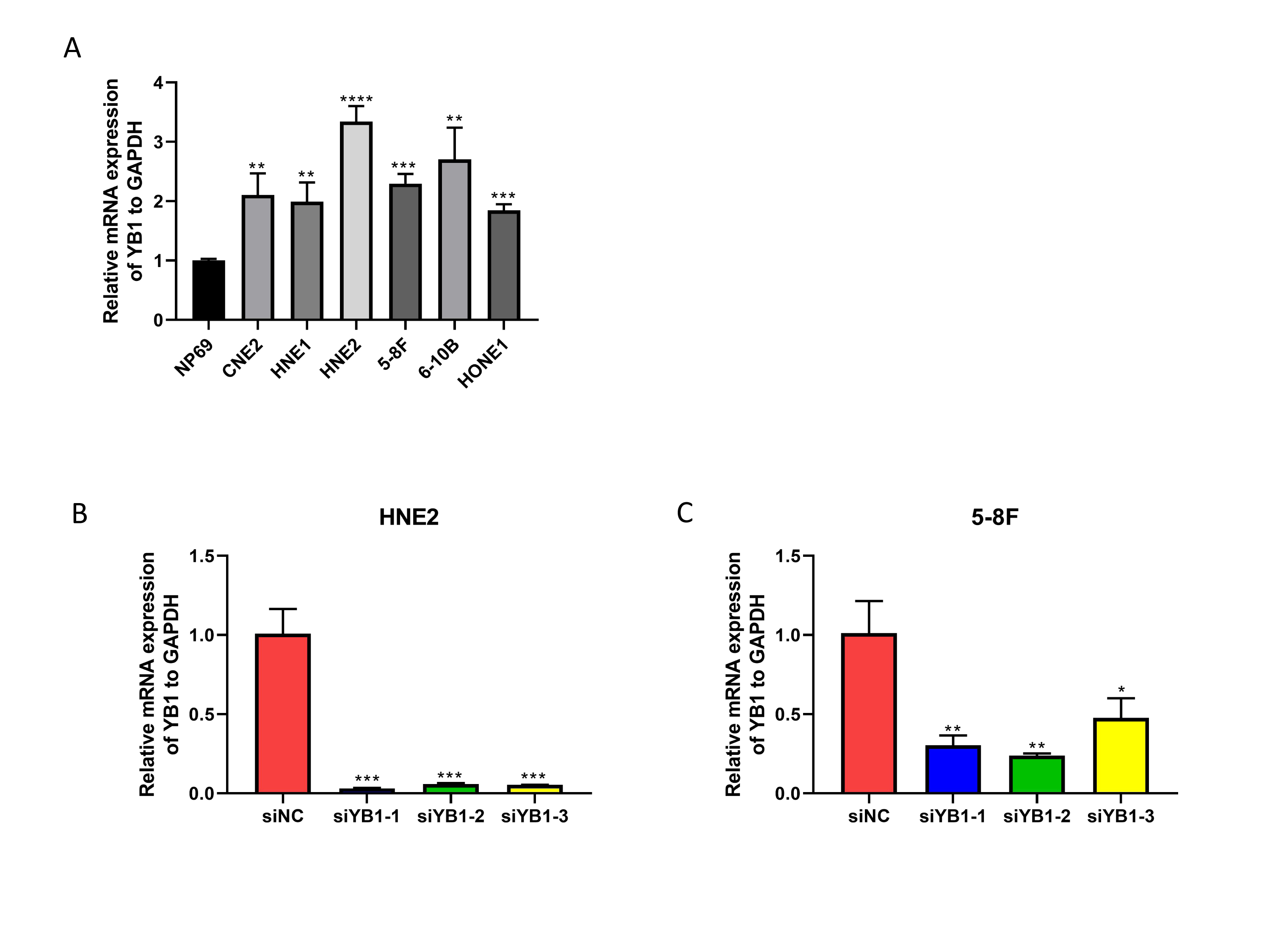

Supplement: Supplementary file 2 — Supplementary Figure 1. [file 41598_2022_7636_MOESM2_ESM.jpg]

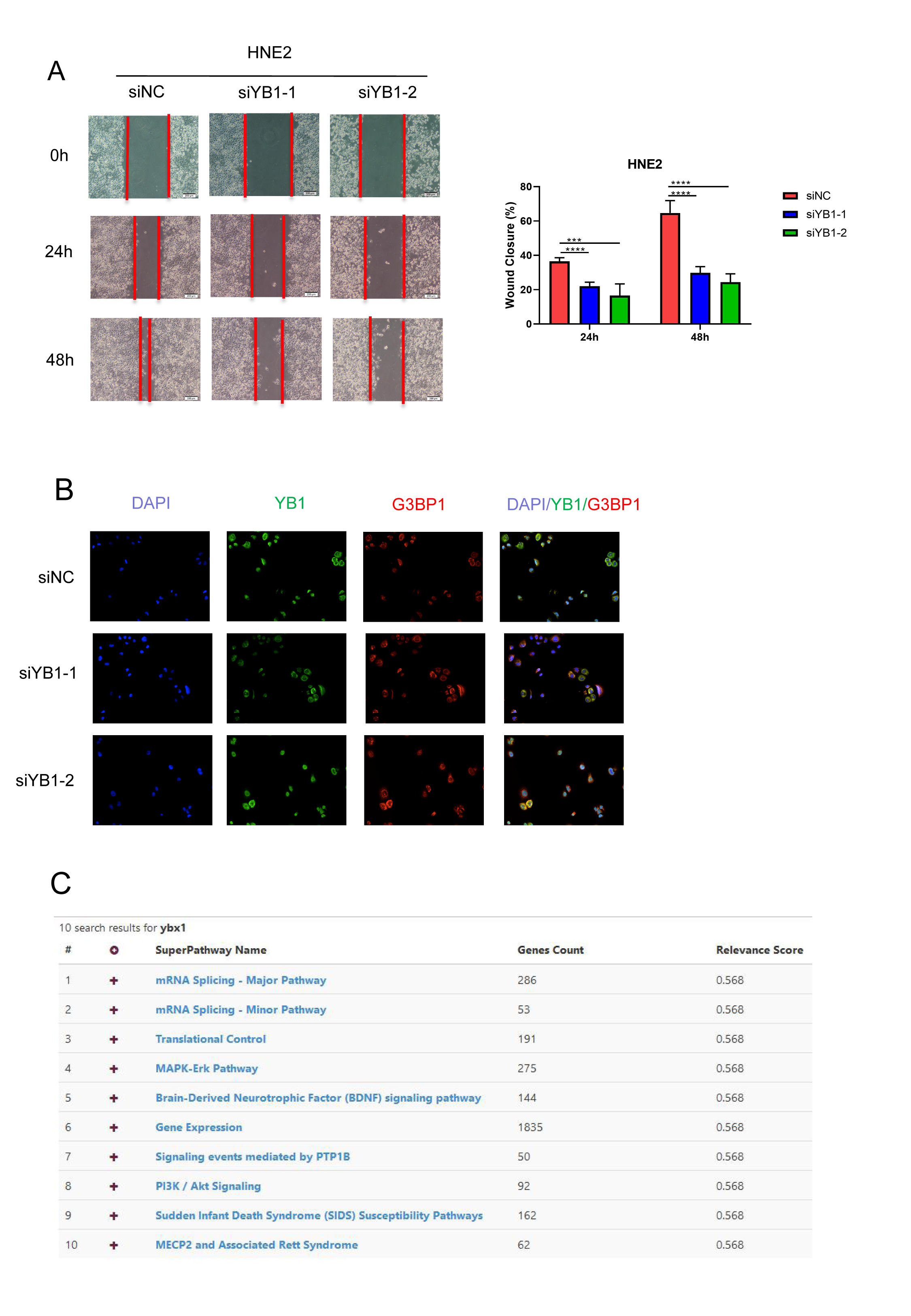

Supplement: Supplementary file 3 — Supplementary Figure 2. [file 41598_2022_7636_MOESM3_ESM.jpg]
