# Supplementary material for: YB1 associates with oncogenetic roles and poor prognosis in nasopharyngeal carcinoma
Source: Sci Rep. 2022 Mar 8;12:3699. doi: 10.1038/s41598-022-07636-z (PMC8904596; doi:10.1038/s41598-022-07636-z)
Supplement: Supplementary file 4 — Supplementary Table 1. [file 41598_2022_7636_MOESM4_ESM.doc]

**Supplementary Table 1: Significant changes of YBX1 expression in transcription level between HNSC and normal control tissues (ONCOMINE database).**

| Types of *HNSC* VS. *Control* | Fold Change | P value | t-test | Ref |
| --- | --- | --- | --- | --- |
| Tongue Carcinoma | 2.242 | 1.05E-9*** | 8.220 | Pyeon Multi-cancer Statistics ^[1]^ |
| Oropharyngeal Carcinoma | 2.015 | 1.36E-6*** | 6.408 | Pyeon Multi-cancer Statistics ^[1]^ |
| Oral Cavity Carcinoma | 2.059 | 2.29E-4*** | 5.546 | Pyeon Multi-cancer Statistics ^[1]^ |
| Floor of the Mouth Carcinoma | 2.749 | 6.42E-6*** | 7.643 | Pyeon Multi-cancer Statistics ^[1]^ |
| Tonsillar Carcinoma | 1.712 | 0.002** | 3.692 | Pyeon Multi-cancer Statistics ^[1]^ |
| Head and Neck Squamous Cell Carcinoma | 1.644 | 6.26E-4*** | 5.171 | Cromer Head-Neck Statistics ^[2]^ |
| Nasopharyngeal Carcinoma | 1.556 | 1.36E-6*** | 5.787 | Sengupta Head-Neck Statistics ^[3]^ |
| Oral Cavity Squamous Cell Carcinoma | 1.637 | 0.001** | 3.935 | Toruner Head-Neck Statistics ^[4]^ |

****p* < 0.05, ***p* < 0.01, ****p* < 0.001**

**Reference:**

[1] Pyeon D, Newton MA, Lambert PF, et al. Fundamental differences in cell cycle deregulation in human papillomavirus-positive and human papillomavirus-negative head/neck and cervical cancers. *Cancer Res*. 2007;67(10):4605-4619. doi:10.1158/0008-5472.CAN-06-3619.

[2] Cromer A, Carles A, Millon R, Ganguli G, Chalmel F, Lemaire F, Young J, Dembélé D, Thibault C, Muller D, Poch O, Abecassis J, Wasylyk B. Identification of genes associated with tumorigenesis and metastatic potential of hypopharyngeal cancer by microarray analysis. Oncogene. 2004 Apr 1;23(14):2484-98.

[3] Sengupta S, den Boon JA, Chen IH, Newton MA, Dahl DB, Chen M, Cheng YJ, Westra WH, Chen CJ, Hildesheim A, Sugden B, Ahlquist P. Genome-wide expression profiling reveals EBV-associated inhibition of MHC class I expression in nasopharyngeal carcinoma. Cancer Res. 2006 Aug 15;66(16):7999-8006.

[4] Toruner GA, Ulger C, Alkan M, Galante AT, Rinaggio J, Wilk R, Tian B, Soteropoulos P, Hameed MR, Schwalb MN, Dermody JJ. Association between gene expression profile and tumor invasion in oral squamous cell carcinoma. Cancer Genet Cytogenet. 2004 Oct 1;154(1):27-35.
